# Impact of Chromosomal Fusion and Transposable Elements on the Genomic Evolution and Genetic Diversity of *Ilex* Species

**DOI:** 10.3390/plants13182649

**Published:** 2024-09-21

**Authors:** Zhenxiu Xu, Haikun Wei, Mingyue Li, Yingjie Qiu, Lei Li, Ke-Wang Xu, Zhonglong Guo

**Affiliations:** 1 Co-Innovation Center for Sustainable Forestry in Southern China, College of Life Sciences, Nanjing Forestry University, Nanjing 210037, China; xu123xzx@njfu.edu.cn (Z.X.); weihaikun@njfu.edu.cn (H.W.); 2 Peter O’Donnell Jr. School of Public Health, University of Texas Southwestern Medical Center, 5323 Harry Hines Blvd, Dallas, TX 75390, USA; 3 National Key Laboratory of Wheat Improvement, Peking University Institute of Advanced Agricultural Sciences, Shandong Laboratory of Advanced Agricultural Sciences at Weifang, Weifang 261000, China

**Keywords:** chromosome fusion, transposable elements, evolution, *Ilex*

## Abstract

The genus *Ilex* belongs to the sole family and is the single genus within the order Aquifoliales, exhibiting significant phenotypic diversity. However, the genetic differences underlying these phenotypic variations have rarely been studied. In this study, collinearity analyses of three *Ilex* genomes, *Ilex latifolia* Thunb., *Ilex polyneura* (Hand.-Mazz.) S. Y. Hu, and *Ilex asprella* Champ. ex Benth., indicated a recent fusion event contributing to the reduction of chromosomes in *I. asprella*. Comparative genome analyses showed slight differences in gene annotation among the three species, implying a minimal disruption of genes following chromosomal fusion in *I. asprella*. Comprehensive annotation of transposable elements (TEs) revealed that TEs constitute a significant portion of the *Ilex* genomes, with LTR transposons being predominant. TEs exhibited an inverse relationship with gene density, potentially influencing gene regulation and chromosomal architecture. TE insertions were shown to affect the conformation and binding sites of key genes such as 7-deoxyloganetin glucosyltransferase and transmembrane kinase (TMK) genes, highlighting potential functional impacts. The structural variations caused by TE insertions suggest significant roles in the evolutionary dynamics, leading to either loss or gain of gene function. This study underscores the importance of TEs in shaping the genomic landscape and evolutionary trajectories of *Ilex* species.

## 1. Introduction

Genetic diversity, the variation in genes among individuals or species, is crucial for the adaptability and survival of species [1,2,3]. Several factors contribute to genetic diversity, including mutation, polyploidy, chromosomal changes, transposition, and more [4,5,6]. For chromosomal changes, chromosomal fusion and fission are widely recognized as primary drivers of the evolution of fundamental chromosome numbers in both the animal and plant kingdoms [7,8,9]. For instance, the origin of human chromosome 2 resulted from the head-to-head fusion of two ancestral ape chromosomes [10]. In *Heliconius* species, the fusion events produced the ten longest chromosomes from ten pairs of shorter progenitors [11]. The transition from mitotic chromosomes to meiotic chromosomes through chromosomal fusion events was observed in both ancient wild *Morus notabilis* C. K. Schneid. and cultivated *Morus alba* L. [12]. In *Artemisia argyi* H. Lév. & Vaniot, the fusion of ancestral 8- and 9-like chromosomes resulted in the formation of chromosome 10, which was accompanied by one inversion and two intrachromosomal translocation events [9]. Chromosome fusions offer valuable insights into the dynamic evolution of genomes and the acquisition of adaptability. On the one hand, this process may lead to substantial changes in genome organization and gene regulation, impacting the evolutionary trajectory of a species [13]. On the other hand, the fusion events can lead to a reduction in chromosome number, potentially resulting in reproductively isolated “chromosomal races” and creating reproductive barriers, thereby driving speciation [11,14,15,16]. In plant genomes, chromosome fusion events are particularly noteworthy for their role in shaping karyotype evolution and contributing to genetic diversity [7,8].

Transposable elements (TEs) are DNA sequences that transpose from one location in the genome to another. These elements were first observed more than 70 years ago by Barbara McClintock [17]. Eukaryotic transposons are categorized into different classes based on their movement strategy. *Class I* consists of RNA transposons or retrotransposons, which can be further classified into two subtypes: long terminal repeat (LTR) and non-LTR retrotransposons [18,19,20,21]. LTR-retrotransposons possess LTRs at the 5′ and 3′ ends, while non-LTR retrotransposons do not. Retrotransposons rely on RNA intermediates that are converted into new copies by reverse transcriptases before being integrated into another position (copy and paste). *Class II* consists of DNA transposons, which transpose directly through transposases [20,21]. The terminal inverted repeats (TIRs) are recognized by transposases, which then catalyze the excision of the element from one genomic location to another (cut and paste). More recently, rolling circle elements, such as Helitrons, have been identified as a distinct and abundant group of DNA transposons. Unlike the “cut-and-paste” mechanism, these elements replicate through a “peel-and-paste” mechanism. It is hypothesized that the sense strand is “peeled” off and serves as a template to synthesize a second strand, forming a circular double-stranded DNA intermediate [18,20,21].

To date, the role of TEs in shaping genome function, speciation, genetic diversity, and adaptive variation in plants has been revealed extensively [22,23,24,25]. TEs exert significant effects on the structure and function of plant genomes [26]. In *Arabidopsis thaliana* (L.) Heynh., TEs have been documented to influence coding regions, with *Copia*-like and En/Spm-like sequences being over-represented in exons, leading to changes in gene functions, such as the enrichment of kinase activity or deficiency of structural molecule activity [27]. In the rice and maize genomes, Pack-TYPE TEs capture and recombine coding DNA, resulting in new transcriptional variants and accelerating gene evolution [28]. They trigger gene expression and functional enrichment in various biological processes, such as post-embryonic development, flower development, and morphogenesis, thereby promoting the plasticity of plant genomes. TEs in the *Helitron* superfamily inserted into the 3′ UTR of the *FLOWERING LOCUS C* (*FLC*) gene to reduce its expression level. This has promoted the natural variation in flowering time under different environmental conditions, explaining the high phenotypic diversity of *Capsella rubella* Reut after a genetic bottleneck [29]. In the genome of the apple, a specific *gypsy*-like LTR retrotransposon closely associated with the red fruit skin phenotype was discovered. This element, inserted upstream of *MdMYB1*, a key gene regulating the anthocyanin biosynthesis pathway, promotes the formation of the red fruit skin by enhancing the expression of *MdMYB1* [30]. An active *hAT* in maize altered the translation rate by inserting into the coding sequence of the *ZmSWEET4c* gene and creating allelic variation, reducing protein abundance, and consequently, affecting the size of corn kernels. This study suggested the potential role of transposons in regulating the translation process, contributing to phenotypic diversity and adaptability in plants [31].

By integrating whole-genome analysis and large-scale resequencing, the role of TE insertion polymorphisms in regulating gene expression and the domestication of morphotypes in *Brassica rapa* L. has been revealed. Specific insertions of *Copia* retrotransposons altered the expression and structure of genes such as *BrMYB18.1*, *BrFLOR1.2*, and *BrVRN1.2*, which are associated with the morphological type and flowering time, promoting diversity in *B. rapa* morphotypes and adaptation to various climatic conditions [32]. TE insertions have provided raw materials for the domestication of *Oryza sativa* L. and its wild relative, *Oryza rufipogon* Griff., promoting the formation of new agronomic traits. The expression of genes *OsRbohB* and *LIP19* is affected by PILE insertions, thereby reducing the thousand grain weight of rice and influencing its adaptability to cold and heat environmental stresses [33]. Through comparative analysis of 21 angiosperm species, it was found that miniature inverted-repeat transposable elements (MITEs), especially those from the *Mutator*, *Tc1-Mariner*, and *PIF-Harbinger* superfamilies, are the primary source of new microRNAs (miRNAs). These new miRNAs are formed through a transposition–transcription process and tend to target genes associated with environmental adaptability. For example, the MITE-miRNA Osa-miRN2285 regulates the cold tolerance-related gene *LOC_Os06g39750*, thereby affecting response and adaptability to temperature and other environmental changes in rice [34].

Aquifoliaceae, the sole family within the order Aquifoliales, encompasses a single genus, *Ilex* [35]. Characterized by their vibrant berries and distinctive foliage, *Ilex* species are frequently used as ornamental plants in gardens. These plants are also rich in beneficial compounds, such as terpenes, and are used as teas (e.g., Kudingcha made from *I. latifolia*) and medicinal products [36]. Despite their diverse applications, basic research on *Ilex* species remains relatively scarce. Recently, advances in sequencing technology have successfully assembled the genomes of three *Ilex* species, *Ilex latifolia* Thunb., *Ilex polyneura* (Hand.-Mazz.) S. Y. Hu, and *Ilex asprella* Champ. ex Benth., providing robust data support for comparative genomic studies [37,38,39,40].

Aquifoliaceae encompasses species with distinct botanical and genetic characteristics. Among the *Ilex* species with available genomes, *I. latifolia* and *I. polyneura* are tree-like in form, contrasting with the shrub-like growth habit of *I. asprella*. Fruit coloration at maturity varies, with *I. latifolia* and *I. polyneura* producing red fruits, while *I. asprella* bears black fruits. Genetically, *I. latifolia* and *I. polyneura* share a basic chromosome number of 20 (x = 20), whereas *I. asprella* has 19 chromosomes in the monoploid state (x = 19). Despite the known botanical differences, the genetic diversity at the molecular level among these three *Ilex* species remains poorly understood.

In this study, we employed three assembled genomes in *Ilex* to conduct comparative genomic analyses. Through chromosomal collinearity analysis, we demonstrated that chromosomal fusion has occurred in *I. asprella*, resulting in a reduced basic chromosome number of 19. Comprehensive annotation of TEs revealed that TEs constitute a significant portion of the *Ilex* genomes, potentially influencing gene regulation and protein structures by inserting into gene bodies or promoters. Via these bioinformatic analyses, our results revealed genetic diversity among the three *Ilex* species in terms of their genomic architecture, TE distribution, as well as the *cis*-regulatory elements associated with these TEs, elucidating the impact of chromosomal fusion and TEs on the genomic evolution and genetic diversity among *Ilex* species.

## 2. Results

### 2.1. Karyotype Evolution Driven by Chromosome Fusion

**Figure 1:**
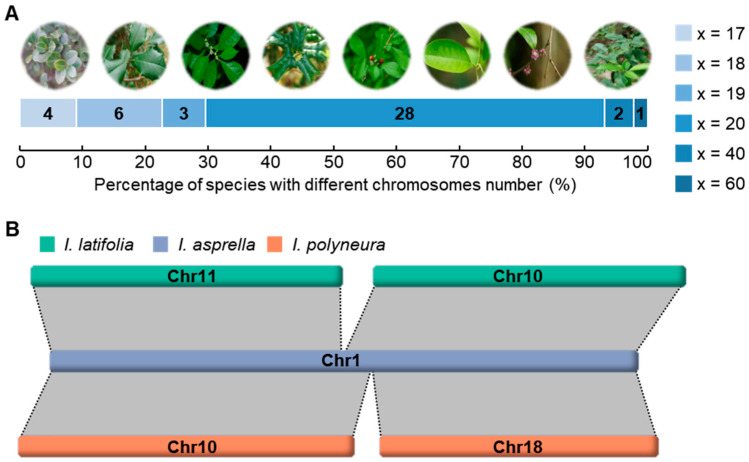
Chromosome number in *Ilex* and chromosome fusion in *I. asprella*. (**A**) Statistics of the chromosome number in representative species of *Ilex*. The photos from left to right indicate *I. crenata* Thunb. (x = 17), *I. opaca* Aiton and *I. verticillata* (L.) A. Gray (x = 18), *I. cornuta* Lindl. & Paxton (x = 19), *I. decidua* Walter, *I. godajam* (Colebr.) Wall. ex Hook.f. and *I. pubescens* Hook. & Arn. (x = 20), and *I. pedunculosa* (x = 60). (**B**) Collinearity plot showing the fusion of Chr1 in *I. asprella*.

To gain insights into karyotype evolution, we surveyed the chromosome numbers in species of the Aquifoliaceae family. Chromosome number records for 44 species were retrieved from the Chromosome Counts Database (https://taux.evolseq.net/CCDB_web/search/, accessed on 26 February 2024), a community resource for plant chromosome numbers [41]. These records reveal that 28 of the 44 species (63.6%) contain 20 chromosomes in monoploid state (x = 20), which is the most representative karyotype. There are some exceptions, including six species (13.6%) that contain 18 chromosomes in monoploid state, four species (9.1%) contain 17 chromosomes in monoploid state, and three species (6.8%) contain 19 chromosomes in monoploid state. Additionally, we found that *I. anomala* Hook. & Arn. and *I. argentina* Lillo have 40 chromosomes, while *I. pedunculosa* Miq. has 60 chromosomes (Figure 1A and Table S1). Despite only a limited number of species being available, these data provide a glimpse into the diversity of the basic chromosome number in Aquifoliaceae.

**Figure 2:**
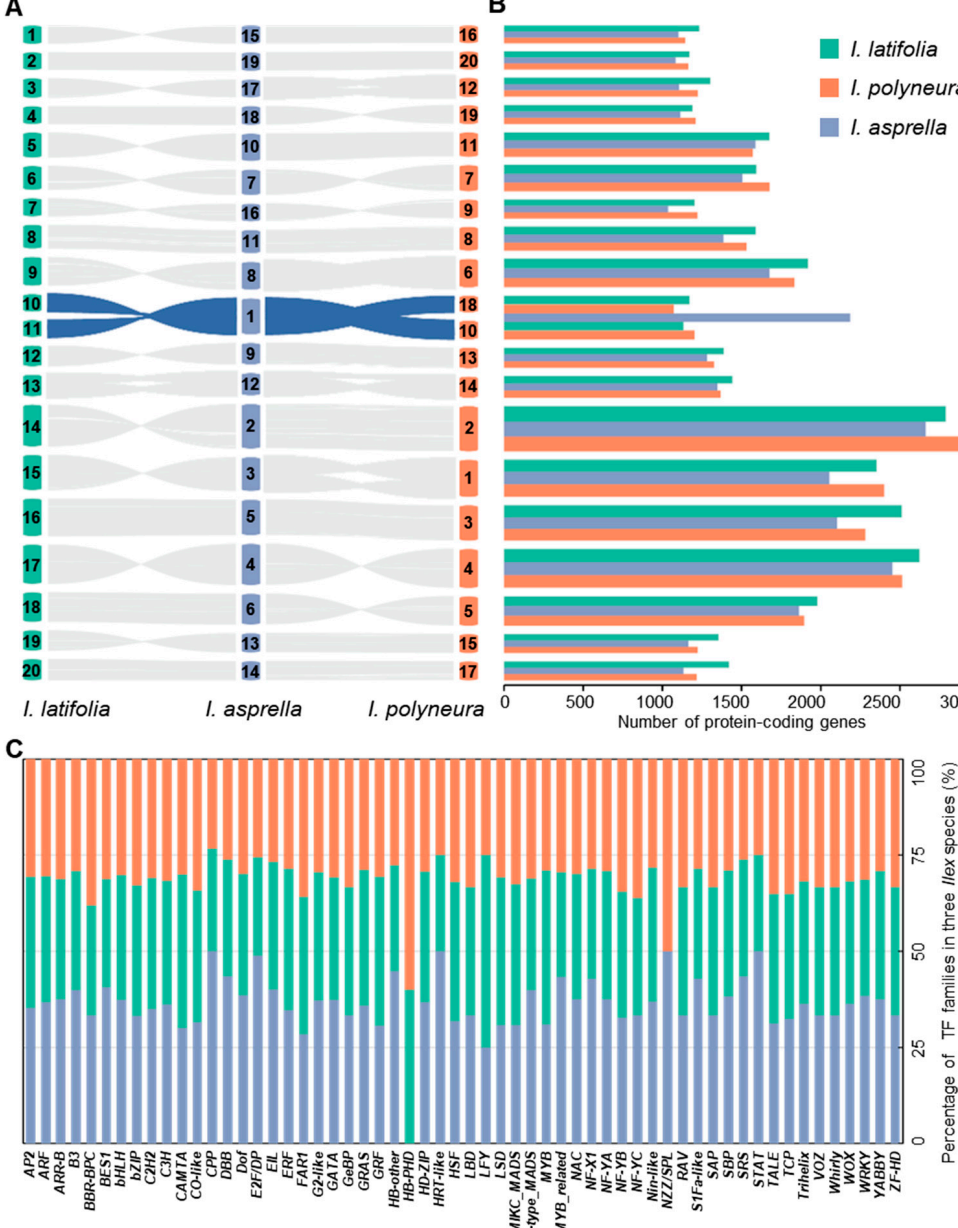
Comparative genomic analyses in three *Ilex* species. (**A**) Collinearity analysis in *I. latifolia*, *I. polyneura*, and *I. asprella*. The chromosome fusion in *I. asprella* is highlighted in blue. (**B**) The number of annotated protein-coding genes in corresponding chromosomes of *I. latifolia*, *I. polyneura*, and *I. asprella*. (**C**) Statistics of 58 transcription factor families in three *Ilex* species. Percentages in *I. latifolia*, *I. polyneura*, and *I. asprella* are marked in green, orange, and blue, respectively.

Given the doubled or tripled chromosome counts in *I. anomala*, *I. argentina*, and *I. pedunculosa* compared with the canonical counts in Aquifoliaceae, we proposed that these changes were the result of recent diploidization or triploidization events. Additionally, 13 of the 44 species (29.5%) harbor 17 to 19 chromosomes in the monoploid state. To further explore the evolutionary forces driving the reduction of chromosomes in these species, we performed collinearity analyses among three available *Ilex* genomes. Among these, *I. latifolia* and *I. polyneura* have 20 chromosomes, while *I. asprella* has 19 chromosomes in the monoploid state. Our results suggested that chromosomes Chr10 and Chr11 in *I. latifolia*, as well as Chr10 and Chr18 in *I. polyneura*, exhibited a high degree of synteny with Chr1 in *I. asprella* (Figure 1B and Figure 2A). The length of Chr1 in *I. asprella* is approximately 54 million base pairs (Mbp), which is comparable to the sum of the two syntenic chromosomes in *I. latifolia* (~57 Mbp) and *I. polyneura* (~56 Mbp). Considering the unassembled sequences on telomeres, we did not delve into the reason for the relatively shorter chromosome in *I. asprella* following chromosome fusion. According to previous phylogenetic studies [42], the divergence of *I. latifolia* occurred prior to the divergence of *I. polyneura* and *I. asprella*. Therefore, our findings demonstrated that the missing chromosome in *I. asprella* is caused by a recent chromosome fusion event, after its divergence from *I. polyneura*.

### 2.2. Comparative Genome Reveals Slight Differences in Genes Annotation

Through collinearity analyses of entire genomes, our results exhibited one-to-one alignment of 18 chromosomes and 1 (or 2) fused chromosome among the three *Ilex* species (Figure 2A and Figures S1–S3) [43]. We then compared the number of protein-coding genes (hereinafter referred to as genes) among the three *Ilex* species. A total of 97,057 genes were identified, including 33,043 in *I. latifolia*, 31,990 in *I. polyneura*, and 29,839 in *I. asprella* (Figure 2B and Tables S2 and S3). Mapping these genes to individual chromosomes, we found that the number of genes in twelve chromosomes exhibited a coincident tendency with the total gene numbers in the three species. In addition, the number of genes from five chromosomes was highest in *I. polyneura*. As for the fused chromosomes, we detected 2185 genes located in the Chr1 of *I. asprella*, while 1173 genes of Chr10 and 1134 genes of Chr11 from *I. latifolia*, as well as 1202 genes of Chr10 and 1071 genes of Chr18 from *I. polyneura* were detected. The number of genes after chromosome fusion was slightly reduced compared to *I. latifolia* (−122 genes) and *I. polyneura* (−88 genes). This finding revealed that chromosome fusion in *I. asprella* caused minimal disruption to pre-existing genes.

Then, we identified totals of 2134, 2005, and 1823 transcription factors (TFs) in *I. asprella*, *I. latifolia*, and *I. polyneura* using PlantTFDB [44], respectively. A statistical analysis of their numbers in each family showed that the majority of families exhibited similar content across the three species, except for two TF families, *NZZ/SPL* and *HB-PHD* (Figure 2C and Table S4). The *NZZ/SPL* TF family, which is crucial for the early development of microsporangia and involved in the initial stages of archesporial cell proliferation and differentiation, is notably absent in the genome of *I. latifolia*. The *HB-PHD* TF family, which plays a vital role in modulating plant resistance to heavy metals, is absent in the genome of *I. asprella*. The potential absence of these two gene families in the *Ilex* species could be correlated with species-specific adaptive traits, reflecting evolutionary adjustments to their respective environments or ecological niche. Alternatively, these absences might also be a consequence of the incomplete genome assemblies, where some regions have not yet been sufficiently resolved, resulting in the omission of these gene families in the current genomes. Further investigation is required to confirm the absence of these TF families.

### 2.3. Identification of TEs in Three Ilex Species

**Figure 3:**
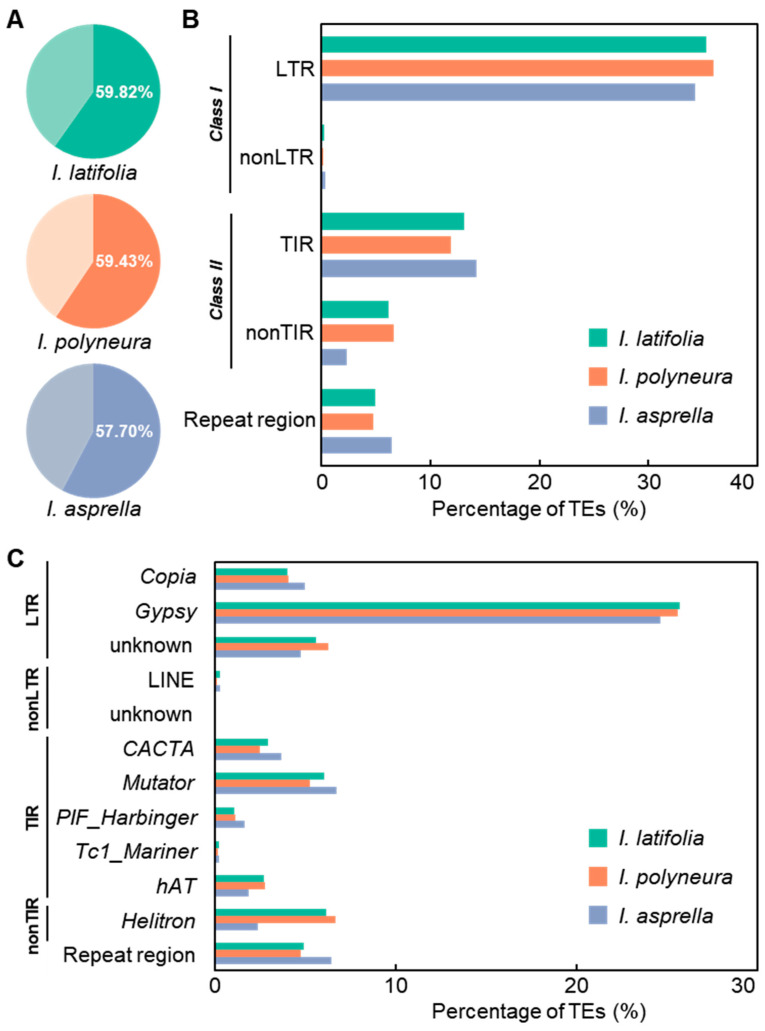
Identification of TEs in three *Ilex* genomes. (**A**) Pie charts showing the total proportion of TEs in the genomes of *I. latifolia*, *I. polyneura*, and *I. asprella.* (**B**,**C**) Bar charts showing the proportion of TEs belonging to *Class I*, *Class II* (**B**), and each superfamily (**C**) in the three genomes.

We performed a comprehensive annotation of TEs in the three *Ilex* species [45]. Our results indicated that TEs account for 59.82% of the genome in *I. latifolia*, 59.43% in *I. polyneura*, and 57.7% in *I. asprella* (Figure 3A). Transposons are known to categorize into *Class I* (retrotransposons) and *Class II* (DNA transposons). TEs in *Class I* include LTR (long terminal repeat) and non-LTR elements, while *Class II* includes TIR (terminal inverted repeat) and non-TIR elements. Among the TEs, LTR transposons constituted the highest proportion in the *Ilex* genomes, with 35.3% in *I. latifolia*, 35.96% in *I. polyneura*, and 34.34% in *I. asprella* (Figure 3B and Table 1). TIR transposons followed, accounting for 13.12% in *I. latifolia*, 11.87% in *I. polyneura*, and 14.24% in *I. asprella* (Figure 3B and Table 1). This classification and identification of TEs revealed that these two main types of transposons are proportionately represented across all three species.

Further classification of transposon superfamilies within the different types of transposons showed that the *Gypsy* superfamily is the most predominant across all three species, accounting for 25.69% in *I. latifolia*, 25.59% in *I. polyneura*, and 24.63% in *I. asprella* (Figure 3C and Table 1). Within the LTR transposons, the *Gypsy* superfamily is more than four times as abundant as the *Copia* superfamily. In the TIR transposons, the *Mutator* superfamily is predominant, followed by the *CACTA* superfamily (Figure 3C and Table 1). These results suggest a consistency in the primary types of transposon families across the three species, despite variations in their specific distribution. The high proportion of *Gypsy* elements within LTR transposons and the prominence of the *Mutator* superfamily within TIR transposons highlight the conserved nature of these elements in shaping the genomic landscape of the *Ilex* species.

### 2.4. TEs Mediate Genetic Effects in Three Ilex Species

**Figure 4:**
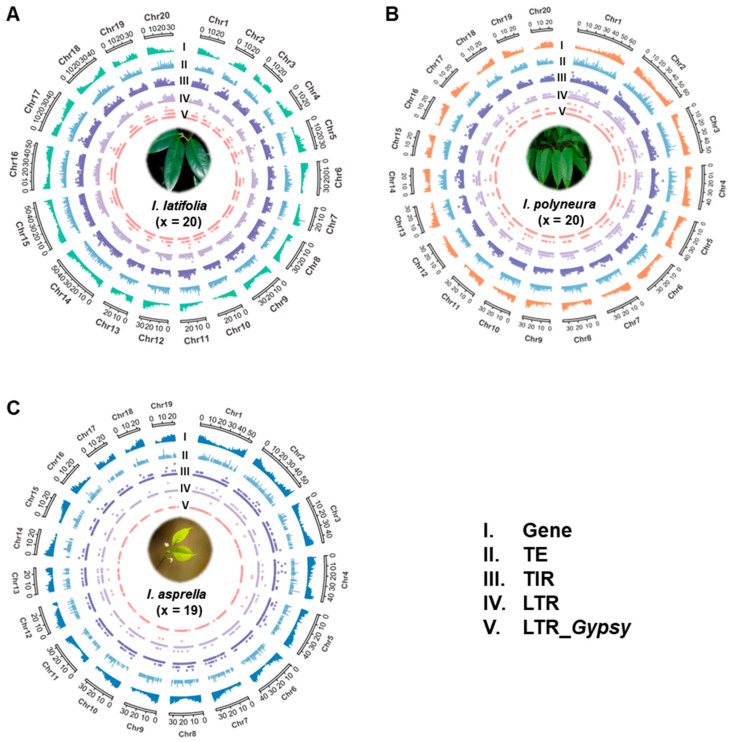
Distribution of protein-coding genes and TEs in genomes of three *Ilex* species. (**A**–**C**) Three circular plots represent the densities of distinct genomic features in *I. latifolia* (**A**), *I. polyneura* (**B**), and *I. asprella* (**C**). Layers of circular plots from outside to inside indicate (I) gene, (II) transposable elements, (III) terminal inverted repeat, (IV) long terminal repeat, and (V) LTR_*Gypsy*. The ideogram scale is in Mbp.

To explore the potential genetic effects of TEs, we analyzed the distribution density of genes, TEs, TIRs, LTRs, and *Gypsy* elements on the chromosomes of *I. latifolia*, *I. polyneura*, and *I. asprella*, respectively (Figure 4A–C and Figures S4–S6) [46]. Our analyses revealed a generally uniform distribution of genes across the chromosomes of all three *Ilex* species. However, a notable inverse relationship between TE density and gene density was observed. As the density of TEs increased, the density of genes tended to decrease. This pattern was consistent across the genomes of *I. latifolia*, *I. polyneura*, and *I. asprella*, suggesting a potential regulatory or structural impact of TEs on gene distribution. These findings are instrumental in understanding the chromosomal architecture and genetic landscape of these *Ilex* species. The observed TE distributions and their relationship with gene densities provide insights into the evolutionary dynamics and regulatory mechanisms shaping the genomes of *I. latifolia*, *I. polyneura*, and *I. asprella*.

**Figure 5:**
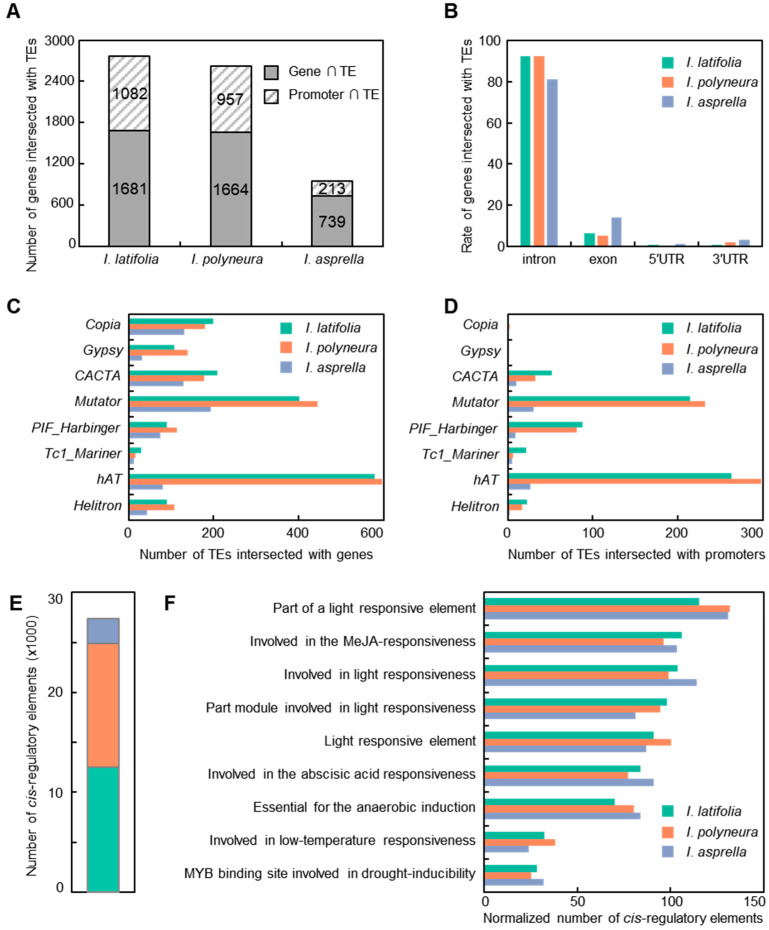
Analysis of protein-coding genes potentially affected by TEs. (**A**) Number of protein-coding genes intersect with TEs. The solid and dashed rectangles indicate TEs intersect with gene bodies and promoters, respectively. Promoters are defined as the 2000 bps upstream of the transcriptional start site. (**B**) Proportion of genes which contain TEs in exons, introns, 5′ UTR, and 3′ UTR regions. (**C**,**D**) Number of TEs in distinct superfamilies which intersect with gene bodies (**C**) and promoters (**D**). (**E**) Number of predicted *cis*-regulatory elements caused by TE insertions in promoters. (**F**) The functional annotation of *cis*-regulatory elements caused by TE insertions in promoters. The normalized number is calculated by the number of one type of element divided by the total number of elements, then multiplied by 1000.

We further identified genes potentially influenced by TEs [47]. Our results revealed that 2763, 2621, and 952 genes contained TEs in promoters or gene bodies in *I. latifolia*, *I. polyneura*, and *I. asprella*, respectively (Figure 5A and Table S5). The number of TE-regulated genes in *I. asprella* was significantly less than that in the other two species. Among these, 1681 genes in *I. latifolia*, 1664 genes in *I. polyneura*, and 739 genes in *I. asprella* harbored TEs in their gene bodies. Examination of the 2000 bps upstream of the transcriptional start sites revealed 1082, 957, and 213 TE-regulated promoters in *I. latifolia*, *I. polyneura*, and *I. asprella*, respectively. The numbers of TE-regulated promoters in *I. latifolia* and *I. polyneura* were about five times that of *I. asprella*. As for TEs located in gene bodies, our results suggested that most TEs overlapped with introns, accounting for 92.2% in *I. latifolia*, 92.4% in *I. polyneura*, and 81.2% in *I. asprella* (Figure 5B and Table S5). A small number of genes contained TEs in the 5′ untranslated region (5′ UTR) and 3′ untranslated region (3′ UTR). Additionally, 6.3% of genes in *I. latifolia*, 5.2% of genes in *I. polyneura*, and 14.0% of genes in *I. asprella* were intersected with exons. These genes were reasonably considered as loss-of-function-resembling T-DNA insertions or frameshift mutations. These observations indicated notable differences in the regulation of genes by TEs among the three *Ilex* species.

A total of 4181 TEs were identified intersecting with gene bodies, with 1707 TEs in *I. latifolia*, 1774 TEs in *I. polyneura*, and 700 TEs in *I. asprella* (Figure 5C and Table S6). This indicated a reduced number of TEs overlapping with gene bodies in *I. asprella*. Categorization of these TEs revealed that the *hAT* superfamily was the most prominent gene regulators in the genomes of *I. latifolia* and *I. polyneura*, followed by the *Mutator* superfamily. In contrast, the *Mutator* superfamily predominates in affecting gene bodies in *I. asprella*, followed by the *Copia* and *CACTA* superfamilies. Among the three species, 1419 TEs were identified to intersect with promoters, specifically 663 TEs in *I. latifolia*, 670 TEs in *I. polyneura*, and 86 TEs in *I. asprella* (Figure 5D and Table S7). These data consistently revealed a lower quantity of TEs overlapping with promoters in *I. asprella*. The *Mutator* and *hAT* superfamilies were the two most prevalent overlapping with promoters in *I. asprella*, whereas in *I. latifolia* and *I. polyneura*, the *hAT* superfamily predominated, followed by the *Mutator* superfamily. The *Copia* and *Gypsy* superfamilies were exceedingly rare across all three species. Our analysis demonstrated substantial disparities in the genetic influence of TEs among the three *Ilex* species. *I. asprella* consistently exhibited a reduced number of TEs overlapping with both gene bodies and promoters compared to *I. latifolia* and *I. polyneura*, reflecting distinct regulatory roles and evolutionary pressures.

The analyses of *cis*-regulatory elements within TEs that intersect with promoters identified a total of 27,364 elements in the three species, specifically 12,545 in *I. latifolia*, 12,328 in *I. polyneura*, and 2491 in *I. asprella* (Figure 5E and Table S8) [48]. The number of *cis*-regulatory elements associated with TEs in *I. latifolia* and *I. polyneura* were similar and approximately five times higher than that in *I. asprella*. The higher abundance of these elements in *I. latifolia* and *I. polyneura* compared to *I. asprella* suggested different evolutionary pressures and functional significances. We then annotated these elements using a bioinformatic method. To mitigate the potential misleading effects caused by quantity variance, we normalized the number of *cis*-regulatory elements and selected the top nine functions based on their frequency (Figure 5F and Table S8). Our results indicated that elements related to light responsiveness were significantly enriched in all three species. Other enriched functions included responses to MeJA, abscisic acid, low temperatures, and drought, highlighting the critical role of TE insertions in plant defense mechanisms, and hormone and stress responses.

### 2.5. Impact of TE Insertions on Protein Structures: Two Case Studies

**Figure 6:**
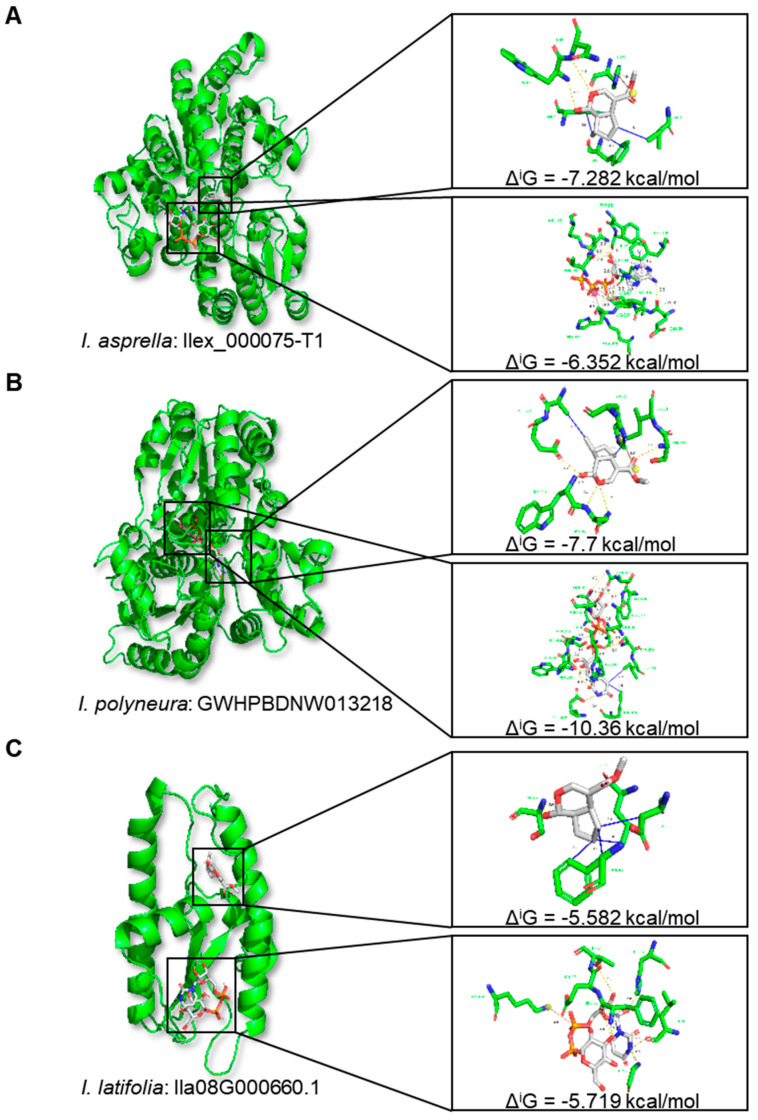
Simulated docking structures of 7−deoxyloganetin glucosyltransferase with two small-molecule ligands, 7−deoxyloganetin and UDP−alpha−D−glucose. Protein structures of 7−deoxyloganetin glucosyltransferase in *I. asprella* (**A**)*, I. polyneura* (**B**), and *I. latifolia* (**C**) are indicated in green. Ball and stick structures with red, blue, and gray indicate 7-deoxyloganetin (**top**) and UDP−alpha−D−glucose (**bottom**). TEs belonging to *Gypsy* superfamily insert into the exons of *Ila08G000660.1* in *I. latifolia*. Δ^i^G (kcal/mol) indicates the binding free energy at the interface.

The analysis of TE−mediated genes identified one 7−deoxyloganetin glucosyltransferase gene (*Ila08G000660.1*) in *I. latifolia* that had a TE insertion within an exon belonging to the *Gypsy* superfamily. Its orthologous genes in *I. asprella* (*Ilex_000075-T1*) and *I. polyneura* (*GWHPBDNW013218*) did not overlap with TEs. Previous studies have shown that 7-deoxyloganetin glucosyltransferase (EC 2.4.1.324) can bind to two small−molecule ligands, 7−deoxyloganetin and UDP−alpha−D−glucose, simultaneously [49,50]. Our docking simulations revealed the significant differences in the conformation of the orthologous genes in *I. asprella* (Figure 6A) and *I. polyneura* (Figure 6B) compared to *Ila08G000660.1* in *I. latifolia* (Figure 6C). Additionally, the binding sites of the 7-deoxyloganetin glucosyltransferases with the two ligands were distinct among the three species.

We further calculated the binding free energy at the interface, denoted as Δ^i^G (kcal/mol), to assess the molecular docking capacity between the 7-deoxyloganetin glucosyltransferase and the two ligands. Our findings showed that the Δ^i^G at the interface between Ilex_000075-T1 and 7-deoxyloganetin was −7.282 kcal/mol, and between Ilex_000075-T1 and UDP-alpha-D-glucose in *I. asprella* was −6.352 kcal/mol (Figure 6A and Table S9). The Δ^i^G at the interface between GWHPBDNW013218 and 7−deoxyloganetin was −7.7 kcal/mol, and between GWHPBDNW013218 and UDP−alpha−D-glucose in *I. polyneura* was −10.36 kcal/mol (Figure 6B and Table S9). However, the Δ^i^G at the interface between Ila08G000660.1 and 7-deoxyloganetin was −5.582 kcal/mol, and between Ila08G000660.1 and UDP−alpha−D−glucose in *I. latifolia* was −5.719 kcal/mol (Figure 6C and Table S9). Given the inverse relationship between docking capacity and the Δ^i^G value, our data suggested that the binding ability of Ila08G000660.1 in *I. latifolia* was disrupted by insertion of the *Gypsy* superfamily.

**Figure 7:**
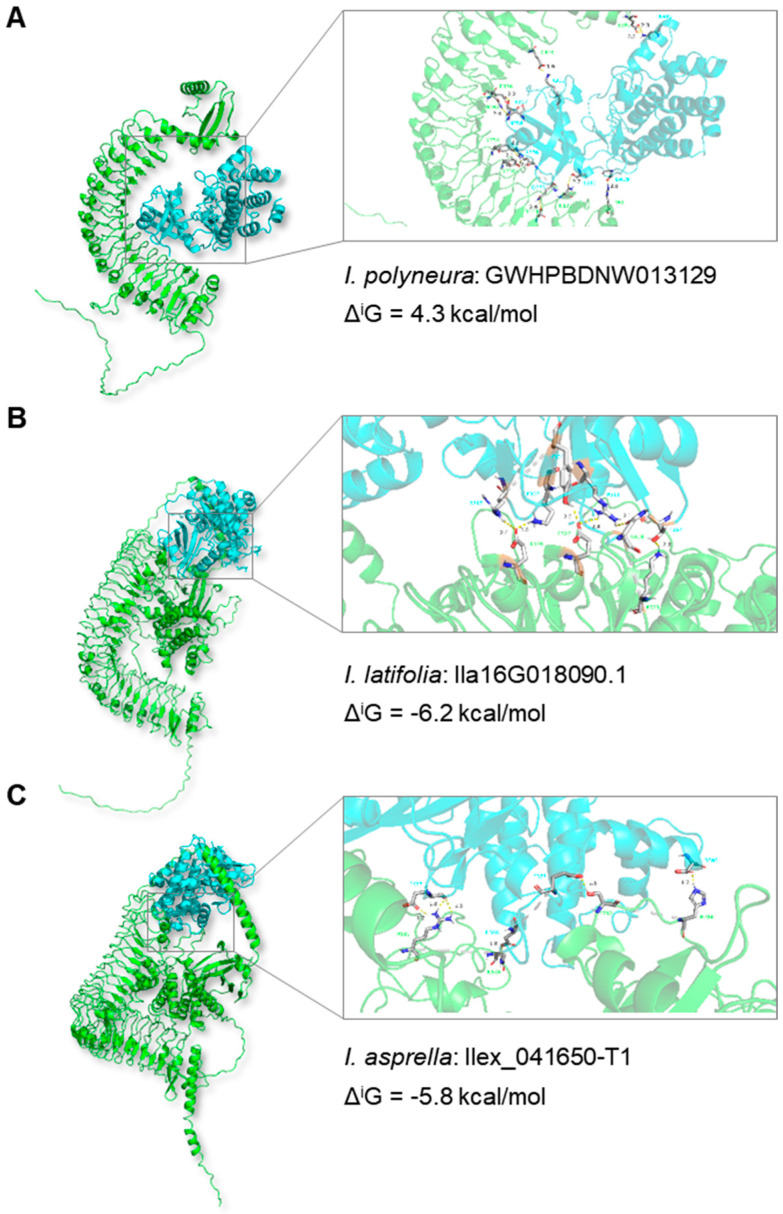
Simulated binding structures of TMK acceptors and their donors. Three TMK proteins, in *I. polyneura* (**A**)*, I. latifolia* (**B**), and *I. asprella* (**C**), interactions with donors (AtMAK1, At4g33430) are predicted by AlphaFold3 [53]. Green structures indicate TMK proteins, while blue structures indicate donors. TEs belonging to *CACTA* superfamily insert into the exons of *TMK* gene in *I. latifolia* and *I. asprella*. Δ^i^G (kcal/mol) indicates the binding free energy at the interface.

Another case involved the *transmembrane kinase* (*TMK*) gene in *I. polyneura* (*GWHPBDNW013129*), which did not overlap with TEs. However, in *I. latifolia* (*Ila16G018090.1*) and *I. asprella* (*Ilex_041650-T1*), TEs belonging to the *CACTA* superfamily have inserted into the exons of the *TMK* genes. Previous studies in *Arabidopsis* demonstrated the interaction between TMK protein and its donor, AtBAK1 (At4g33430) [51,52]. We simulated the binding structures of TMK proteins in the three *Ilex* species and their donor, AtBAK1, using AlphaFold3 [53]. Our results indicated that the binding conformation and position between TMK and AtBAK1 in *I. polyneura* (Figure 7A) were quite different from those in *I. latifolia* (Figure 7B) and *I. asprella* (Figure 7C). Calculation of binding free energy at the interface indicated the affinity between TMK and AtBAK1. Our findings revealed that the Δ^i^G at the interface between TMK and AtBAK1 in *I. polyneura* was 4.3 kcal/mol, whereas in *I. latifolia* and *I. asprella* it was −6.2 kcal/mol and −5.8 kcal/mol, respectively (Figure 7A–C and Table S10). Given the inverse relation between interaction capacity and the value of Δ^i^G, our data suggested that the acquisition of the binding ability in *Ilex* may occur after the insertion of the *CACTA* superfamily. Taken together, these two cases highlighted that the structural variations caused by TE insertions may result in potential functional impacts, leading to either loss of function or gain of function.

## 3. Discussion

Our study provides significant insights into the karyotype evolution, genetic diversity, and regulatory mechanisms of *Ilex* species driven by chromosomal fusion and TEs. The survey of chromosome numbers in the Aquifoliaceae family revealed a predominant karyotype with a basic chromosome number of 20, along with variations indicating recent diploidization or triploidization events. Collinearity analyses demonstrated that the fusion of chromosomes in *I. asprella* has minimal disruption on gene content, suggesting a recent chromosomal fusion event after the divergence with *I. polyneura*.

TEs are present across all phyla, exhibiting species-specific differences in their characteristics, prevalence, and functionality. For instance, in *Saccharomyces cerevisiae*, TEs make up only 3% of the genome [54], whereas they can account for as much as 80% in maize [55]. In this study, the comprehensive annotation of TEs across the three *Ilex* species highlighted that TEs account for 57.70% to 59.82% of their genomes. LTR transposons were predominant, followed by TIR transposons, with the *Gypsy* and *Mutator* superfamilies being the most prevalent. The inverse relationship between TE density and gene density across the chromosomes indicates that TEs may play a critical role in gene regulation and chromosomal architecture. This pattern was consistent in *I. latifolia*, *I. polyneura*, and *I. asprella*, suggesting potential regulatory or structural impacts of TEs on genes.

Our findings on the *cis*-regulatory elements within TEs that intersect with promoters identified substantial differences among the three species. *I. latifolia* and *I. polyneura* had a significantly higher number of *cis*-regulatory elements associated with TEs compared to *I. asprella*, suggesting different evolutionary pressures and functional significances. Elements related to light responsiveness, MeJA responsiveness, abscisic acid responsiveness, low-temperature responsiveness, and drought stress were significantly enriched, underscoring the critical role of TE insertions in plant defense mechanisms, hormone responses, and stress responses.

Furthermore, the analysis of TE-mediated genes identified significant differences in the binding interactions of key proteins. For instance, the 7-deoxyloganetin glucosyltransferase gene in *I. latifolia*, which had a TE belonging to *Gypsy* superfamily insertion in its exon, showed disrupted binding ability with its ligands compared to its orthologs in *I. asprella* and *I. polyneura*. On the contrary, the *TMK* genes in *I. latifolia* and *I. asprella*, affected by TE belonging to *CACTA* superfamily insertions, exhibited an increased affinity with their donor AtBAK1 compared to *I. polyneura*, which had no TE insertions. These structural variations caused by TE insertions resulted in potential functional impacts, leading to either loss of function or gain of function.

In short, our study highlights the significant role of chromosomal fusion and TE insertions in shaping the genomic landscape, regulatory mechanisms, and functional diversity of *Ilex* species. The insights gained from this research provide a robust foundation for future molecular studies and enhance our understanding of the evolutionary dynamics of the genome and adaptive strategies of plants.

## 4. Materials and Methods

### 4.1. Source of Genomes and Chromosome Counts

The genomes and corresponding annotations of *I. latifolia* [37], *I. polyneura* [38], and *I. asprella* [39] are available in previously published studies. The files with Fasta and GFF3 format were downloaded from HollyGTD (https://hollygdb.com/, accessed on 26 February 2024), a comprehensive database dedicated to collecting multi-omics data in Aquifoliaceae [40]. Chromosome numbers used in this study were retrieved from the Chromosome Counts Database (https://taux.evolseq.net/CCDB_web/search/, accessed on 26 February 2024) [41].

### 4.2. Collinearity-Based Chromosome Fusion Analysis

MCScanX is a bioinformatics tool used to detect and analyze chromosomal synteny, facilitating the identification and comparison of genomic homologies across different species [43]. In this study, we utilized MCScanX (Python version) to perform a collinearity-based chromosome fusion analysis in three *Ilex* species. The process began with the collection and preparation of chromosomal sequence data from the species involved in the study. We then employed the Python script of MCScanX to perform multiple sequence alignments for identifying homologous regions. The alignment outcomes were analyzed to determine the syntenic relationships between the chromosomes. Based on the synteny analysis results, we constructed the synteny plot to visualize homologous relationships and investigate chromosome fusion events.

### 4.3. Identification of Transcription Factor Families

The PlantTFDB (https://planttfdb.gao-lab.org/, accessed on 25 March 2024) is a database designed for the prediction of plant transcription factors [44]. It allows for the identification of transcription factors by directly inputting protein or nucleic acid sequences. In this study, we utilized PlantTFDB (Version 5.0) to identify transcription factor families in three *Ilex* genomes. We navigated to the PlantTFDB website then accessed the “prediction” tool. Using the default settings, we submitted protein sequences from the three *Ilex* species to predict and annotate their transcription factors.

### 4.4. Identification of Transposable Elements

EDTA (Extensive De-Novo TE Annotator) is a comprehensive bioinformatics tool for identifying transposable elements in plants [45]. It provides complete and high-quality annotations of transposable elements in newly assembled plant genomes by integrating various software and algorithms. In this study, we followed the EDTA software pipeline with default parameters to perform a de novo identification and statistical analysis of retrotransposons and DNA transposons in the genomes of three *Ilex* species.

### 4.5. Distribution of Genes and TEs

We used TBtools-II (Version 2.096) to analyze the distribution characteristics of protein-coding genes and transposable elements within the genome [46]. Firstly, the “Fasta Stats” tool was employed to calculate the lengths of the chromosomes. Following this, the “Table Row Manipulate” tool was used to determine the distribution density of genes and TEs across the genome. Lastly, the “Advanced Circos” tool was applied to visualize the results obtained from the previous steps.

### 4.6. Intersection of Genes and TEs

Bedtools is a collection of algorithmic tools for genomic data analysis, allowing for intersecting, merging, counting, complementing, and format transformation of genomic data [47]. “Bedtools intersect” is a standalone tool within Bedtools designed to calculate the positional relationships between two or more files. In this study, we utilized “Bedtools intersect” to perform an intersection analysis between genes and transposable elements. The regions of genes were further divided into promoter, exon, intron, 5′ UTR, and 3′ UTR. We set parameters as “-f 0.50” to ensure that at least 50% of the transposable elements were covered.

### 4.7. Extraction of Intron Positions in Genome

The standalone tool “Bedtools subtract” from the Bedtools toolkit was used to obtain the location data for introns [47]. The command was executed as “bedtools subtract -a gene.gff -b exon.gff > intron.gff”. The output file was sorted by chromosome position. Subsequently, the “Bedtools merge” tool was applied to consolidate overlapping entries within the intron.sort.gff file, with the command “bedtools merge -i intron.sort.gff -c 4,5 -o distinct > intron.sort.final.gff”.

### 4.8. Identification of Cis-Regulatory Elements

PlantCARE (https://bioinformatics.psb.ugent.be/webtools/plantcare/html/, accessed on 7 May 2024) is a specialized database and online toolkit for analyzing plant *cis*-regulatory elements, providing comprehensive information on various regulatory elements such as enhancers and silencers [48]. The DNA sequences of transposable elements located on promoters were submitted to the PlantCARE website to predict *cis*-regulatory elements. The annotations for each element were also retrieved from the PlantCARE database.

### 4.9. Prediction of Protein Structure

Protein sequences were extracted and formatted into Fasta files. These sequences served as input data for homology modeling using AlphaFold3 (https://golgi.sandbox.google.com/, accessed on 10 June 2024) [53]. The AlphaFold3 algorithm employs deep learning techniques to predict the most likely 3D conformation of a protein, selecting the optimal conformation as the result.

### 4.10. Protein–Protein Interaction Simulation

Information for the ligand At4g33430 was retrieved from the RCSB Protein Data Bank (https://www.rcsb.org/, accessed on 10 June 2024) [56,57]. Corresponding PDB format files were identified and downloaded for subsequent analysis. Protein–protein interaction was performed using the HADDOCK web server (https://rascar.science.uu.nl/haddock2.4/, accessed on 15 June 2024) [58]. The predicted structure of the target protein, along with the ligand protein structures obtained from the PDB, were used as input. The HADDOCK server generated various possible docking poses based on the input structures and interaction constraints. The optimal model was selected from the balanced-order models.

The complex structural model was created and visualized using the PyMOL Molecular Graphics System (Version 3.0.0) [59]. Colors were applied to label the different chains of the two proteins to facilitate visualization and interpretation. The capacity of protein–protein binding was assessed using the PDBePISA server (https://www.ebi.ac.uk/pdbe/pisa/, accessed on 15 June 2024) [60].

### 4.11. Molecular Docking Simulation of Proteins and Small-Molecule Ligands

The small-molecule ligands 7-deoxyloganetin (Compound CID: 10262598) and UDP-alpha-D-glucose (Compound CID: 8629) were obtained from PubChem (https://pubchem.ncbi.nlm.nih.gov, accessed on 19 July 2024) [61]. Corresponding SDF format files were downloaded for subsequent analyses. The SDF files were optimized using the Open Babel and AutoDockTools software (Version 1.5.7), and subsequently, saved in PDBQT format [62,63]. Concurrently, the protein PDB files were refined using AutoDockTools and saved in PDBQT format. The PDBQT files of the protein receptor and small-molecule ligands were inputted into AutoDock Vina for molecular docking simulations [64,65]. The final results were recorded, and the best model was selected based on these simulations. The complex structural model was generated and visualized using the PyMOL Molecular Graphics System (version 3.0.0) [59]. To enhance clarity, different colors were employed to distinguish between the protein amino acid residues and the small-molecule ligands.

## Figures and Tables

**Table 1:** Statistics of identified TEs in three genomes of *Ilex*.

Order	Superfamily	*I. latifolia*			*I. asprella*			*I. polyneura*		
		Count	Length (bp)	%	Count	Length (bp)	%	Count	Length (bp)	%
LTR	*Copia*	38,219	30,700,831	4.01%	51,998	35,494,928	4.96%	35,857	29,629,464	4.08%
	*Gypsy*	145,246	196,792,508	25.69%	170,245	176,221,772	24.63%	146,734	186,042,503	25.59%
	unknown	74,946	42,916,075	5.60%	54,846	33,952,958	4.75%	83,403	45,770,036	6.29%
Non-LTR	LINE_element	3491	2,077,187	0.27%	3790	2,279,086	0.32%	2605	1,113,338	0.15%
	unknown	570	167,545	0.02%	0	0	0.00%	0	0	0.00%
TIR	*CACTA*	69,302	22,641,095	2.96%	79,124	26,401,225	3.69%	58,667	18,315,040	2.52%
	*Mutator*	141,937	46,577,919	6.08%	139,183	48,341,143	6.76%	119,261	38,338,622	5.27%
	*PIF_Harbinger*	26,367	8,491,650	1.11%	37,818	11,904,229	1.66%	26,525	8,109,161	1.12%
	*Tc1_Mariner*	7196	1,968,434	0.26%	6822	1,886,253	0.26%	4588	1,248,614	0.17%
	*hAT*	56,873	20,727,706	2.71%	34,981	13,398,044	1.87%	54,114	20,263,483	2.79%
Non-TIR	*Helitron*	139,768	47,218,541	6.16%	52,976	16,905,447	2.36%	143,665	48,573,149	6.68%
repeat_region		125,490	37,903,467	4.95%	165,145	45,991,681	6.43%	123,449	34,683,080	4.77%
Total		829,405	458,182,958	59.82%	796,928	412,776,766	57.70%	798,868	432,086,490	59.43%

## Data Availability

The original contributions presented in the study are included in the article/Supplementary Materials, further inquiries can be directed to the corresponding authors.

## Supplementary Materials

The following supporting information can be downloaded at: https://www.mdpi.com/article/10.3390/plants13182649/s1, Table S1: Statistics on the number of chromosomes in *Ilex* species; Table S2: Num. of genes in each chromosome on three *Ilex* species; Table S3: Length of each chromosome across three *Ilex* species; Table S4: Num. of transcription factor families on three *Ilex* species; Table S5: Num. of genes intersected with TEs on different gene elements in three *Ilex* species; Table S6: Num. of TEs intersected with gene in three *Ilex* species; Table S7: Num. of TEs intersected with promoter in three *Ilex* species; Table S8: Num. of *cis*-regulatory elements in three *Ilex* species; Table S9: Autodock vina molecular docking results between proteins and ligand in three *Ilex* species; Table S10: PDBePISA interface result between proteins and BAK1 in three *Ilex* species; Figures S1–S3: Chromosomal collinearity analysis in three *Ilex* species with each other; Figures S4–S6: Distribution of protein-coding genes and TEs in genomes of three *Ilex* species, respectively.

## References

[1] Feulner P.G.D., De-Kayne R. (2017). Genome evolution, structural rearrangements and speciation. J. Evol. Biol..

[2] Hughes A.R., Inouye B.D., Johnson M.T.J., Underwood N., Vellend M. (2008). Ecological consequences of genetic diversity. Ecol. Lett..

[3] Reed D.H., Frankham R. (2003). Correlation between fitness and genetic diversity. Conserv. Biol..

[4] Otto S.P., Whitton J. (2000). Polyploid incidence and evolution. Annu. Rev. Genet..

[5] Levin D.A. (2002). The Role of Chromosomal Change in Plant Evolution.

[6] Wessler S.R. (2006). Transposable elements and the evolution of eukaryotic genomes. Proc. Natl. Acad. Sci. USA.

[7] Schubert I., Lysak M.A. (2011). Interpretation of karyotype evolution should consider chromosome structural constraints. Trends Genet..

[8] Weiss-Schneeweiss H., Schneeweiss G.M., Greilhuber J., Dolezel J., Wendel J.F. (2013). Karyotype Diversity and Evolutionary Trends in Angiosperms. Plant Genome Diversity Volume 2: Physical Structure, Behaviour and Evolution of Plant Genomes.

[9] Miao Y., Luo D., Zhao T., Du H., Liu Z., Xu Z., Guo L., Chen C., Peng S., Li J.X. (2022). Genome sequencing reveals chromosome fusion and extensive expansion of genes related to secondary metabolism in *Artemisia argyi*. Plant Biotechnol. J..

[10] JW I.J., Baldini A., Ward D.C., Reeders S.T., Wells R.A. (1991). Origin of human chromosome 2: An ancestral telomere-telomere fusion. Proc. Natl. Acad. Sci. USA.

[11] Cicconardi F., Lewis J.J., Martin S.H., Reed R.D., Danko C.G., Montgomery S.H. (2021). Chromosome fusion affects genetic diversity and evolutionary turnover of functional loci but consistently depends on chromosome size. Mol. Biol. Evol..

[12] Xuan Y., Ma B., Li D., Tian Y., Zeng Q., He N. (2022). Chromosome restructuring and number change during the evolution of *Morus notabilis* and *Morus alba*. Hortic. Res..

[13] Vara C., Paytuví-Gallart A., Cuartero Y., Álvarez-González L., Marín-Gual L., Garcia F., Florit-Sabater B., Capilla L., Sanchéz-Guillén R.A., Sarrate Z. (2021). The impact of chromosomal fusions on 3D genome folding and recombination in the germ line. Nat. Commun..

[14] Hauffe H.C., Searle J.B. (1998). Chromosomal heterozygosity and fertility in house mice (*Mus musculus domesticus*) from Northern Italy. Genetics.

[15] de Vos J.M., Augustijnen H., Bätscher L., Lucek K. (2020). Speciation through chromosomal fusion and fission in Lepidoptera. Philos. Trans. R. Soc. Lond. B Biol. Sci..

[16] Luo J., Sun X., Cormack B.P., Boeke J.D. (2018). Karyotype engineering by chromosome fusion leads to reproductive isolation in yeast. Nature.

[17] McClintock B. (2012). The origin and behavior of mutable loci in maize. Proc. Natl. Acad. Sci. USA.

[18] Wells J.N., Feschotte C. (2020). A field guide to eukaryotic transposable elements. Annu. Rev. Genet..

[19] Kumar A., Bennetzen J.L. (1999). Plant retrotransposons. Annu. Rev. Genet..

[20] Bourque G., Burns K.H., Gehring M., Gorbunova V., Seluanov A., Hammell M., Imbeault M., Izsvák Z., Levin H.L., Macfarlan T.S. (2018). Ten things you should know about transposable elements. Genome Biol..

[21] Makałowski W., Gotea V., Pande A., Makałowska I., Anisimova M. (2019). Transposable Elements: Classification, Identification, and Their Use As a Tool for Comparative Genomics.

[22] Klein S.J., O’Neill R.J. (2018). Transposable elements: Genome innovation, chromosome diversity, and centromere conflict. Chromosome Res..

[23] Stritt C., Wyler M., Gimmi E.L., Pippel M., Roulin A.C. (2019). Diversity, dynamics and effects of long terminal repeat retrotransposons in the model grass *Brachypodium distachyon*. New Phytol..

[24] Ramakrishnan M., Satish L., Sharma A., Kurungara Vinod K., Emamverdian A., Zhou M., Wei Q. (2022). Transposable elements in plants: Recent advancements, tools and prospects. Plant Mol. Biol. Rep..

[25] Hassan A.H., Mokhtar M.M., El Allali A. (2024). Transposable elements: Multifunctional players in the plant genome. Front. Plant Sci..

[26] Parisod C., Alix K., Just J., Petit M., Sarilar V., Mhiri C., Ainouche M., Chalhoub B., Grandbastien M.A. (2010). Impact of transposable elements on the organization and function of allopolyploid genomes. New Phytol..

[27] Lockton S., Gaut B.S. (2009). The contribution of Transposable Elements to expressed coding sequence in *Arabidopsis thaliana*. J. Mol. Evol..

[28] Gisby J.S., Catoni M. (2022). The widespread nature of Pack-TYPE transposons reveals their importance for plant genome evolution. PLoS Genet..

[29] Niu X.-M., Xu Y.-C., Li Z.-W., Bian Y.-T., Hou X.-H., Chen J.-F., Zou Y.-P., Jiang J., Wu Q., Ge S. (2019). Transposable elements drive rapid phenotypic variation in *Capsella rubella*. Proc. Natl. Acad. Sci. USA.

[30] Zhang L., Hu J., Han X., Li J., Gao Y., Richards C.M., Zhang C., Tian Y., Liu G., Gul H. (2019). A high-quality apple genome assembly reveals the association of a retrotransposon and red fruit colour. Nat. Commun..

[31] Chen G., Wang R., Jiang Y., Dong X., Xu J., Xu Q., Kan Q., Luo Z., Springer N.M., Li Q. (2023). A novel active transposon creates allelic variation through altered translation rate to influence protein abundance. Nucleic Acids Res..

[32] Cai X., Lin R., Liang J., King G.J., Wu J., Wang X. (2022). Transposable element insertion: A hidden major source of domesticated phenotypic variation in *Brassica rapa*. Plant Biotechnol. J..

[33] Li X., Dai X., He H., Lv Y., Yang L., He W., Liu C., Wei H., Liu X., Yuan Q. (2024). A pan-TE map highlights transposable elements underlying domestication and agronomic traits in Asian rice. Natl. Sci. Rev..

[34] Guo Z., Kuang Z., Tao Y., Wang H., Wan M., Hao C., Shen F., Yang X., Li L., Arkhipova I. (2022). Miniature Inverted-repeat Transposable Elements drive rapid microRNA diversification in angiosperms. Mol. Biol. Evol..

[35] Loizeau P.A., Andrews S.V., Spichiger S., Aquifoliaceae R., Kubitzki K. (2016). The families and genera of vascular plants. Flowering Plants. Eudicots.

[36] Yao X., Zhang F., Corlett R.T. (2022). Utilization of the hollies (*Ilex* L. spp.): A review. Forests.

[37] Xu K.W., Wei X.F., Lin C.X., Zhang M., Zhang Q., Zhou P., Fang Y.M., Xue J.Y., Duan Y.F. (2022). The chromosome-level holly (*Ilex latifolia*) genome reveals key enzymes in triterpenoid saponin biosynthesis and fruit color change. Front. Plant Sci..

[38] Yao X., Lu Z., Song Y., Hu X., Corlett R.T. (2022). A chromosome-scale genome assembly for the holly (*Ilex polyneura*) provides insights into genomic adaptations to elevation in southwest China. Hortic. Res..

[39] Kong B.L., Nong W., Wong K.H., Law S.T., So W.L., Chan J.J., Zhang J., Lau T.D., Hui J.H., Shaw P.C. (2022). Chromosomal level genome of *Ilex asprella* and insight into antiviral triterpenoid pathway. Genomics.

[40] Guo Z., Wei J., Xu Z., Lin C., Peng Y., Wang Q., Wang D., Yang X., Xu K.W. (2023). HollyGTD: An integrated database for holly (Aquifoliaceae) genome and taxonomy. Front. Plant Sci..

[41] Rice A., Glick L., Abadi S., Einhorn M., Kopelman N.M., Salman-Minkov A., Mayzel J., Chay O., Mayrose I. (2014). The Chromosome Counts Database (CCDB)—A community resource of plant chromosome numbers. New Phytol..

[42] Yang Y., Jiang L., Liu E.D., Liu W.L., Chen L., Kou Y.X., Fan D.M., Cheng S.M., Zhang Z.Y., Peng H. (2023). Time to update the sectional classification of *Ilex* (Aquifoliaceae): New insights from *Ilex* phylogeny, morphology, and distribution. J. Syst. Evol..

[43] Wang Y., Tang H., DeBarry J.D., Tan X., Li J., Wang X., Lee T.H., Jin H., Marler B., Guo H. (2012). MCScanX: A toolkit for detection and evolutionary analysis of gene synteny and collinearity. Nucleic Acids. Res..

[44] Tian F., Yang D.-C., Meng Y.-Q., Jin J., Gao G. (2019). PlantRegMap: Charting functional regulatory maps in plants. Nucleic Acids. Res..

[45] Ou S., Su W., Liao Y., Chougule K., Agda J.R.A., Hellinga A.J., Lugo C.S.B., Elliott T.A., Ware D., Peterson T. (2019). Benchmarking transposable element annotation methods for creation of a streamlined, comprehensive pipeline. Genome Biol..

[46] Chen C., Chen H., Zhang Y., Thomas H.R., Frank M.H., He Y., Xia R. (2020). TBtools: An integrative toolkit developed for interactive analyses of big biological data. Mol. Plant.

[47] Quinlan A.R., Hall I.M. (2010). BEDTools: A flexible suite of utilities for comparing genomic features. Bioinformatics.

[48] Rombauts S., Dehais P., Van Montagu M., Rouze P. (1999). PlantCARE, a plant *cis*-acting regulatory element database. Nucleic Acids Res..

[49] Nagatoshi M., Terasaka K., Nagatsu A., Mizukami H. (2011). Iridoid-specific Glucosyltransferase from Gardenia jasminoides. J. Biol. Chem..

[50] Asada K., Salim V., Masada-Atsumi S., Edmunds E., Nagatoshi M., Terasaka K., Mizukami H., De Luca V. (2013). A 7-Deoxyloganetic acid glucosyltransferase contributes a key step in secologanin biosynthesis in madagascar periwinkle. Plant Cell.

[51] Li J., Wen J., Lease K.A., Doke J.T., Tax F.E., Walker J.C. (2002). BAK1, an *Arabidopsis* LRR Receptor-like Protein Kinase, interacts with BRI1 and modulates brassinosteroid signaling. Cell.

[52] Sun Y., Li L., Macho A.P., Han Z., Hu Z., Zipfel C., Zhou J.-M., Chai J. (2013). Structural basis for flg22-induced activation of the *Arabidopsis* FLS2-BAK1 immune complex. Science.

[53] Abramson J., Adler J., Dunger J., Evans R., Green T., Pritzel A., Ronneberger O., Willmore L., Ballard A.J., Bambrick J. (2024). Accurate structure prediction of biomolecular interactions with AlphaFold 3. Nature.

[54] Kim J.M., Vanguri S., Boeke J.D., Gabriel A., Voytas D.F. (1998). Transposable Elements and Genome Organization: A comprehensive survey of retrotransposons revealed by the complete *Saccharomyces cerevisiae* senome sequence. Genome Res..

[55] Flavell R.B., Bennett M.D., Smith J.B., Smith D.B. (1974). Genome size and the proportion of repeated nucleotide sequence DNA in plants. Biochem. Genet..

[56] Berman H.M. (2000). The Protein Data Bank. Nucleic Acids Res..

[57] Burley S.K., Bhikadiya C., Bi C., Bittrich S., Chao H., Chen L., Craig P.A., Crichlow G.V., Dalenberg K., Duarte J.M. (2023). RCSB Protein Data Bank (RCSB.org): Delivery of experimentally-determined PDB structures alongside one million computed structure models of proteins from artificial intelligence/machine learning. Nucleic Acids Res..

[58] Honorato R.V., Trellet M.E., Jiménez-García B., Schaarschmidt J.J., Giulini M., Reys V., Koukos P.I., Rodrigues J.P.G.L.M., Karaca E., van Zundert G.C.P. (2024). The HADDOCK2.4 web server for integrative modeling of biomolecular complexes. Nat. Protoc..

[59] Chrödinger L.L.C. The PyMOL Molecular Graphics System, Version 3.0.0; PyMOL. https://pymol.org/.

[60] Krissinel E., Henrick K. (2007). Inference of macromolecular assemblies from crystalline state. J. Mol. Biol..

[61] Kim S., Chen J., Cheng T., Gindulyte A., He J., He S., Li Q., Shoemaker B.A., Thiessen P.A., Yu B. (2023). PubChem 2023 update. Nucleic Acids. Res..

[62] O’Boyle N.M., Banck M., James C.A., Morley C., Vandermeersch T., Hutchison G.R. (2011). Open Babel: An open chemical toolbox. J. Cheminf..

[63] Morris G.M., Huey R., Lindstrom W., Sanner M.F., Belew R.K., Goodsell D.S., Olson A.J. (2009). AutoDock4 and AutoDockTools4: Automated docking with selective receptor flexibility. J. Comput. Chem..

[64] Trott O., Olson A.J. (2009). AutoDock Vina: Improving the speed and accuracy of docking with a new scoring function, efficient optimization, and multithreading. J. Comput. Chem..

[65] Eberhardt J., Santos-Martins D., Tillack A.F., Forli S. (2021). AutoDock Vina 1.2.0: New docking methods, expanded force field, andpython bindings. J. Chem. Inf. Model..

