# Supplementary material for: Impact of Chromosomal Fusion and Transposable Elements on the Genomic Evolution and Genetic Diversity of Ilex Species
Source: Plants (Basel). 2024 Sep 21;13(18):2649. doi: 10.3390/plants13182649 (PMC11435385; doi:10.3390/plants13182649)
Supplement: Supplementary file 1 [file plants-13-02649-s001.zip › plants-3157446-supplementary/SFigures 20240913.pdf]

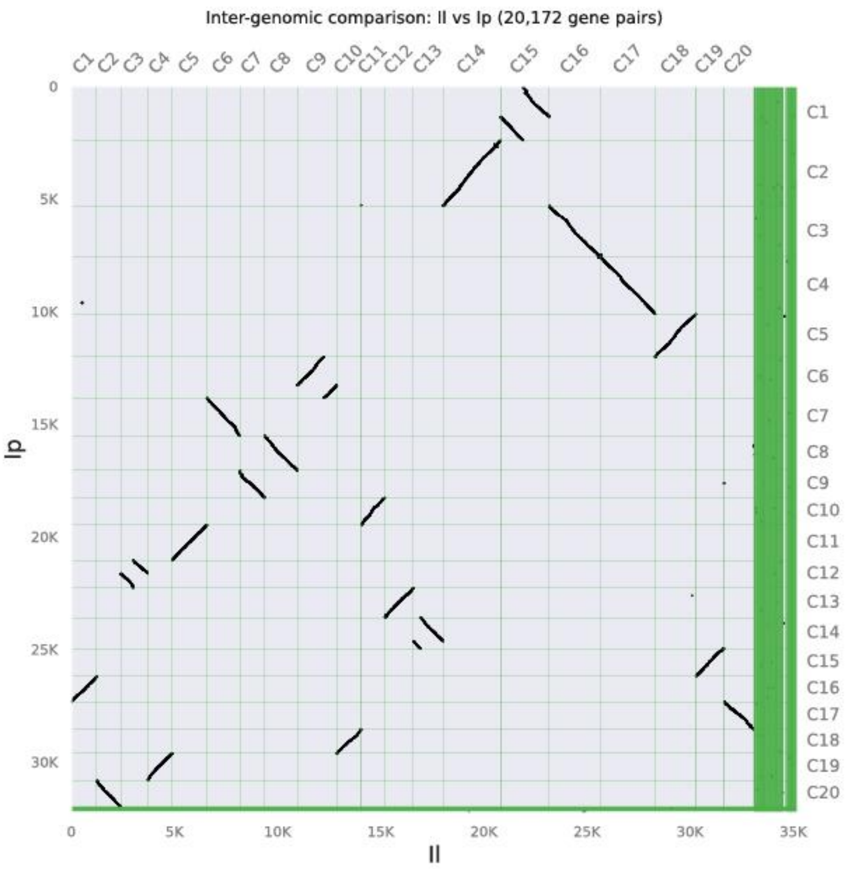

Figure S1. Chromosomal collinearity analysis between *I. latifolia* and *I. polyneura*. A total of 20,172 pairs of high-quality homologous genes were identified. Syntenic relationship was constructed with homologous genes of all chromosomes in *I. latifolia* and *I. polyneura.*

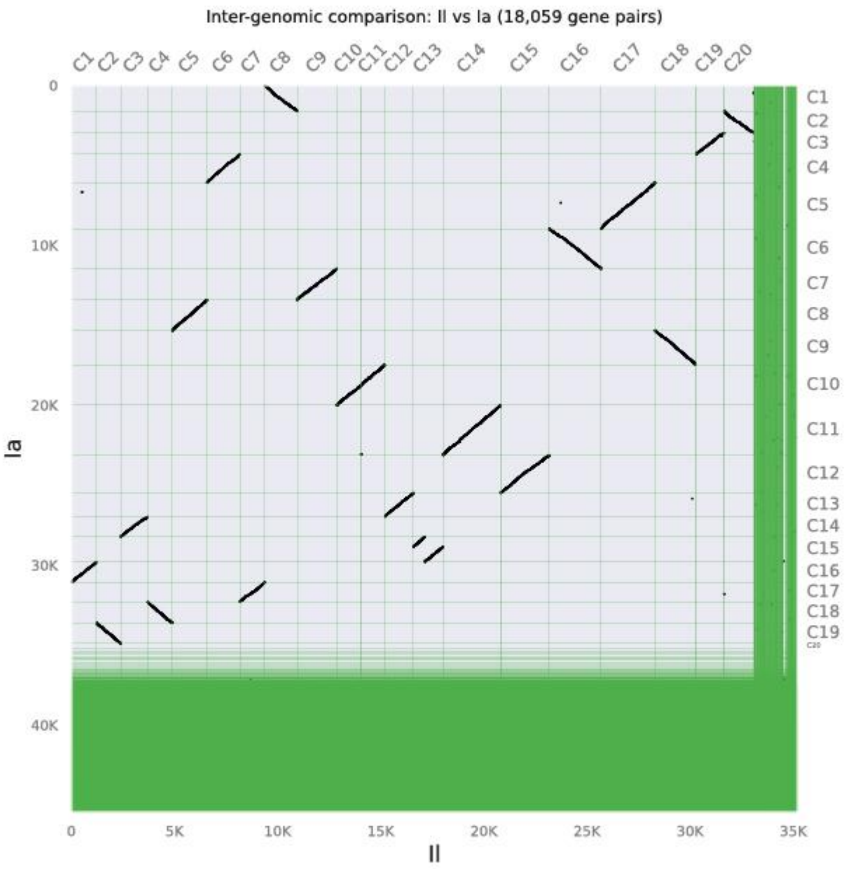

Figure S2. Chromosomal collinearity analysis between *I. latifolia* and *I. asprella*. A total of 18,059 pairs of high-quality homologous genes were identified. Syntenic relationship was constructed with homologous genes of all chromosomes in *I. latifolia* and *I. asprella.*

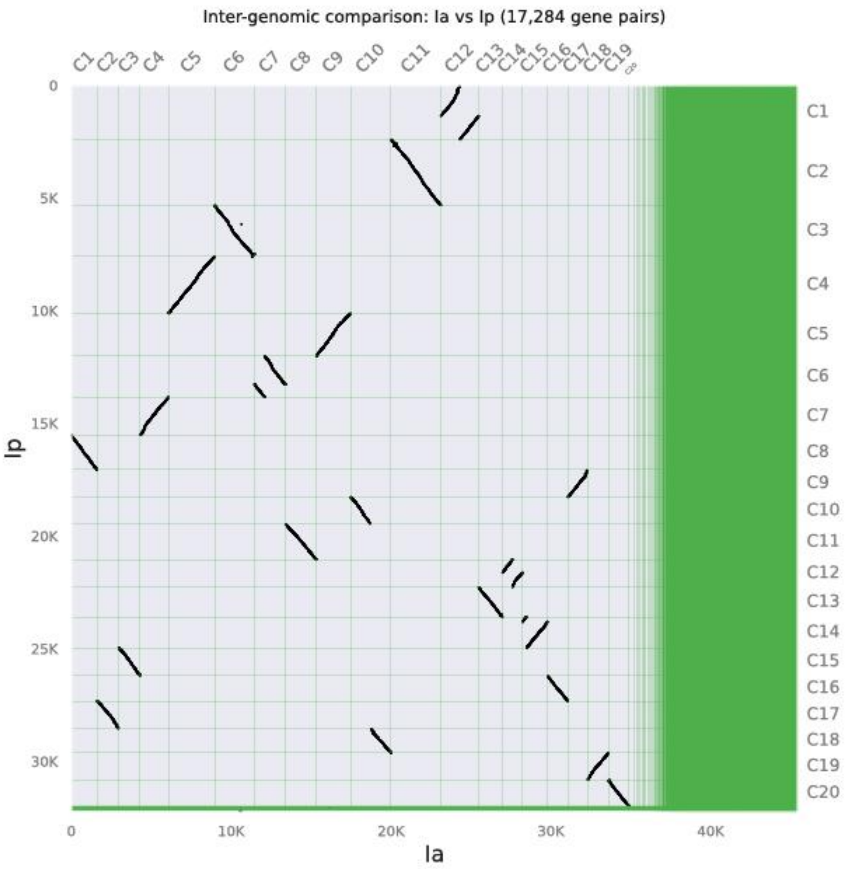

Figure S3. Chromosomal collinearity analysis between *I. asprella* and *I. polyneura*. A total of 17,284 pairs of high-quality homologous genes were identified. Syntenic relationship was constructed with homologous genes of all chromosomes in *I. asprella* and *I. polyneura.*

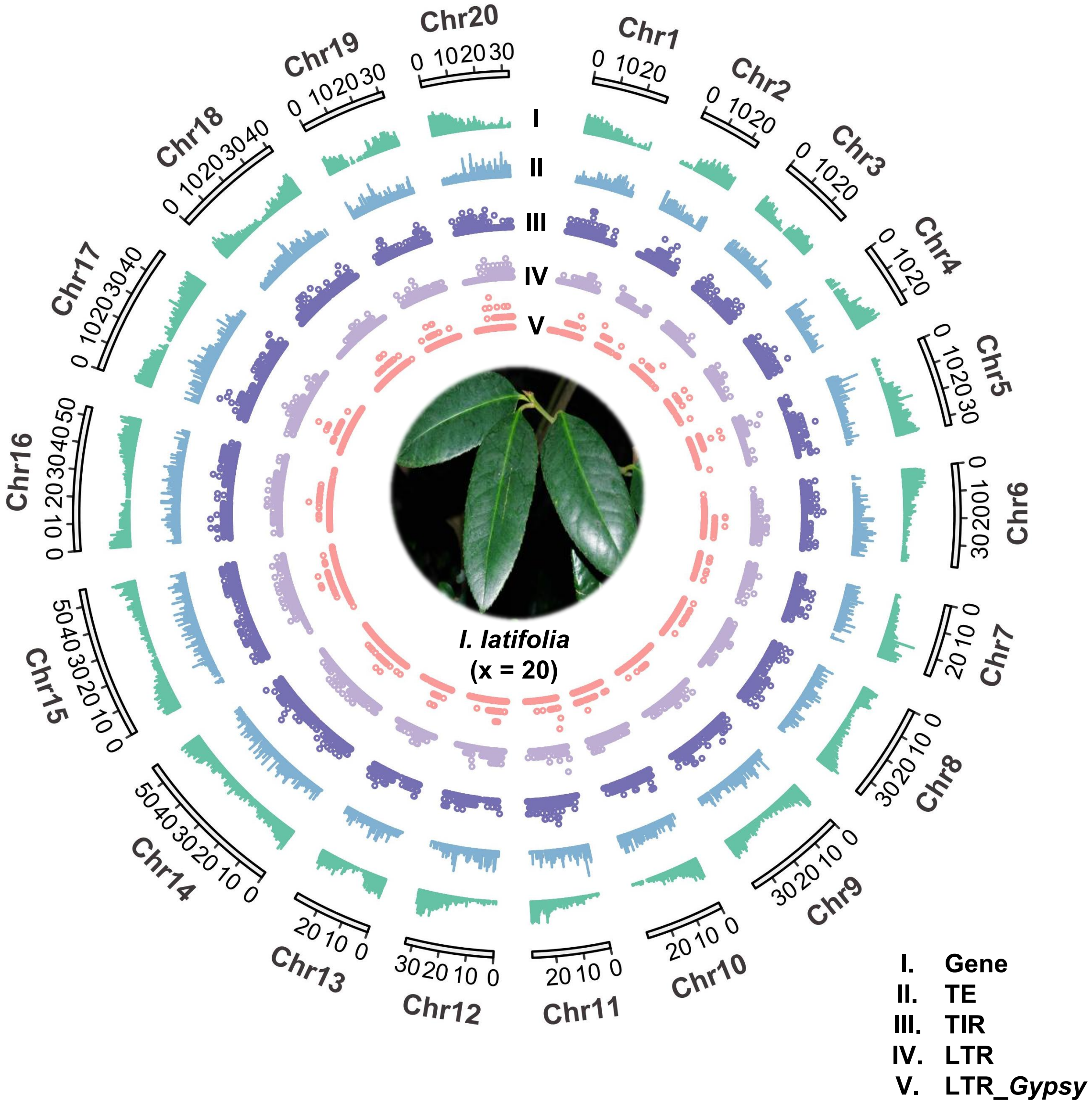

Figure S4. Distribution of protein-coding genes and TEs in *I. latifolia* genome. The layers of circular plots from outside to inside indicate: I) gene, II) Transposable elements, III) Terminal inverted repeat, (IV) Long terminal repeat, and (V) LTR_*Gypsy*. The ideogram scale is in Mbp.

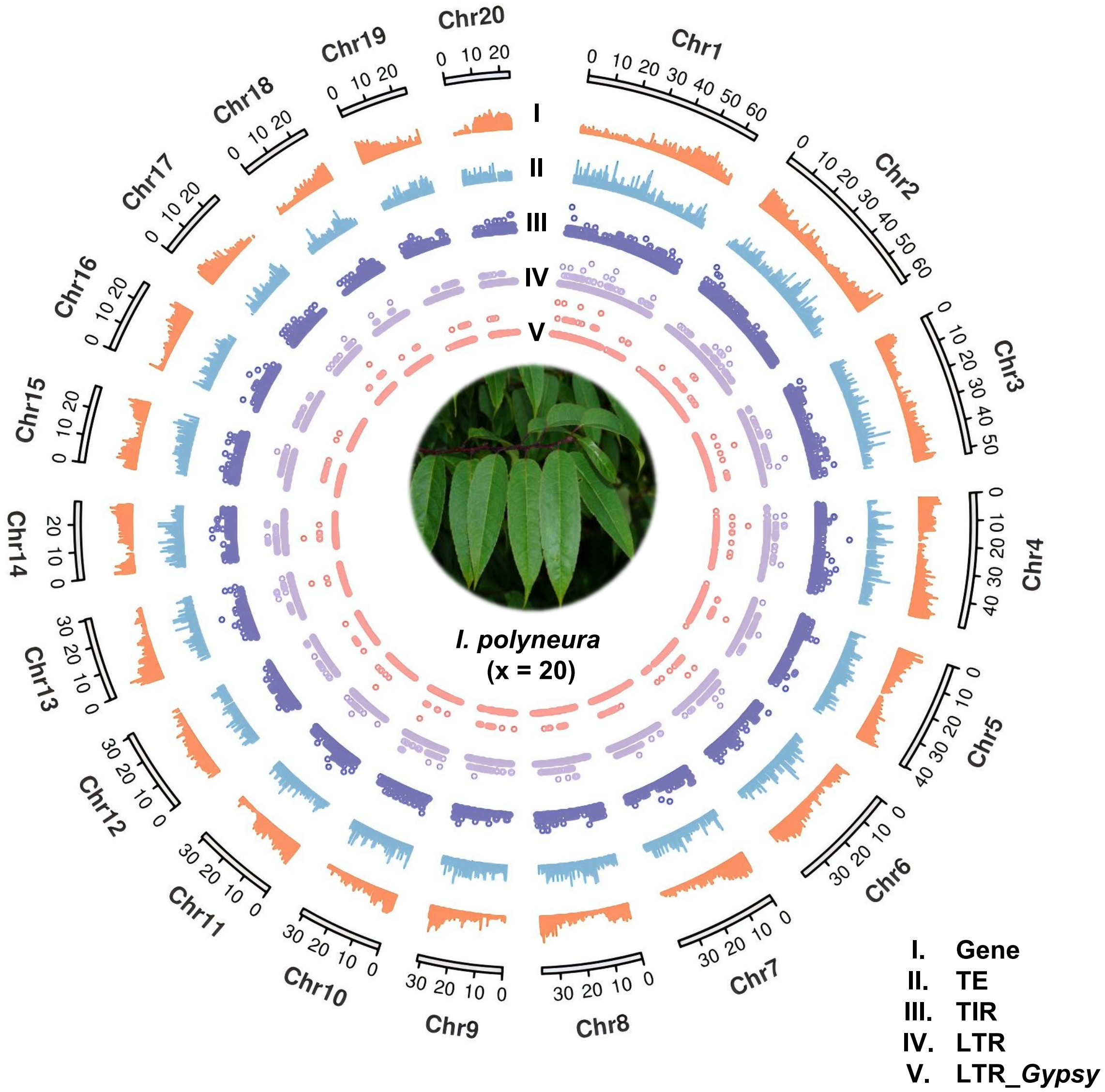

Figure S5. Distribution of protein-coding genes and TEs in *I. polyneura* genome. The layers of circular plots from outside to inside indicate: I) gene, II) Transposable elements, III) Terminal inverted repeat, (IV) Long terminal repeat, and (V) LTR_*Gypsy*. The ideogram scale is in Mbp.

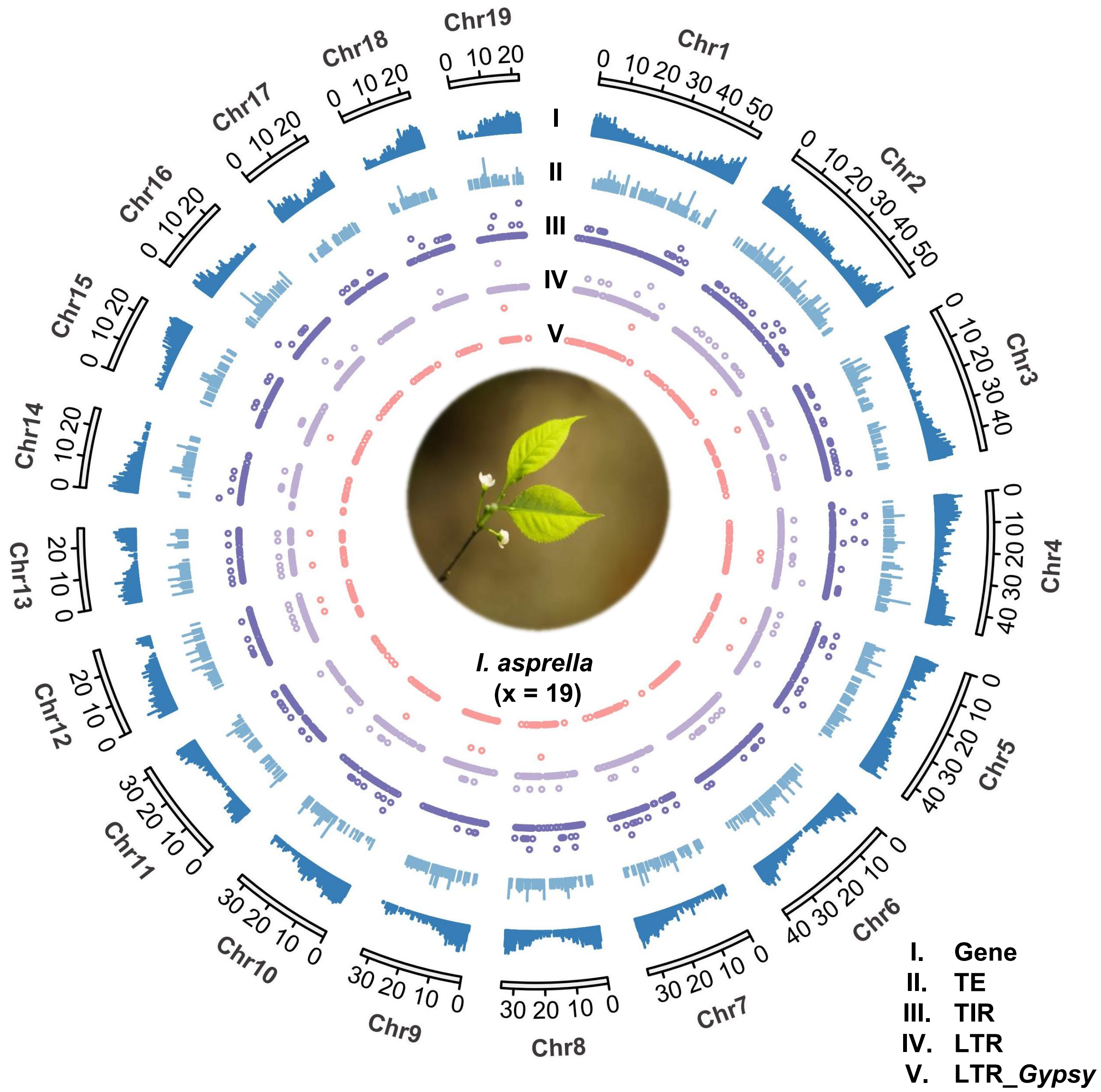

Figure S6. Distribution of protein-coding genes and TEs in *I. asprella* genome. The layers of circular plots from outside to inside indicate: I) gene, II) Transposable elements, III) Terminal inverted repeat, (IV) Long terminal repeat, and (V) LTR_*Gypsy*. The ideogram scale is in Mbp.
